# Speech Emotion Recognition Based on Selective Interpolation Synthetic Minority Over-Sampling Technique in Small Sample Environment

**DOI:** 10.3390/s20082297

**Published:** 2020-04-17

**Authors:** Zhen-Tao Liu, Bao-Han Wu, Dan-Yun Li, Peng Xiao, Jun-Wei Mao

**Affiliations:** 1 School of Automation, China University of Geosciences, Wuhan 430074, China; liuzhentao@cug.edu.cn (Z.-T.L.); wubaohan@cug.edu.cn (B.-H.W.); pampas@cug.edu.cn (P.X.); 1201610733@cug.edu.cn (J.-W.M.); 2 Hubei Key Laboratory of Advanced Control and Intelligent Automation for Complex Systems, Wuhan 430074, China

**Keywords:** speech emotion recognition, data imbalance processing, feature selection, SISMOTE

## Abstract

Speech emotion recognition often encounters the problems of data imbalance and redundant features in different application scenarios. Researchers usually design different recognition models for different sample conditions. In this study, a speech emotion recognition model for a small sample environment is proposed. A data imbalance processing method based on selective interpolation synthetic minority over-sampling technique (SISMOTE) is proposed to reduce the impact of sample imbalance on emotion recognition results. In addition, feature selection method based on variance analysis and gradient boosting decision tree (GBDT) is introduced, which can exclude the redundant features that possess poor emotional representation. Results of experiments of speech emotion recognition on three databases (i.e., CASIA, Emo-DB, SAVEE) show that our method obtains average recognition accuracy of 90.28% (CASIA), 75.00% (SAVEE) and 85.82% (Emo-DB) for speaker-dependent speech emotion recognition which is superior to some state-of-the-arts works.

## 1. Introduction

With the rapid development of human-computer interaction systems, the emotional intelligence has been paid much attention in recent years, by which both the emotional state and implied intentions of the human could be obtained [1]. Primary challenge for realizing human-computer emotional interaction is to identify emotional state of humans accurately and effectively [2,3]. Cues for human emotion recognition can be found from various modalities, including speech, facial expression, and physiological signals, etc. As a main component of emotional intelligence, speech emotion recognition (SER) draws researchers’ attention extensively [4,5].

It is widely accepted that speech conveys not only the semantic meaning but also the emotional information of speakers [6,7]. In recent years, emotions are generally described by discrete models in the form of emotional labels, thus various deep learning architectures were frequently used due to SER being often modeled as a static or dynamic classification problem [8,9,10]. However, most application environments of SER are small sample environments such as patient’s emotional monitoring. A deep learning network that demands massive emotional tagging data has certain limitations in SER [11,12,13], thus SER based on emotional feature engineering and machine learning algorithm plays an important role. Both speaker-dependent (SD) and speaker-independent (SI) SER for small sample environment have attracted much attention [14,15,16], in which SI SER always achieved lower recognition accuracy for small sample environment. In addition, it is time and cost demanding to prepare a certain amount of training data for SD SER, and even more severe in SI SER in some scenarios, e.g., multi-human–multi-robot interaction system in a household environment [17]. Thus, we mainly focus on SD SER in this paper.

As a utility science, the data of SER obtained in the actual application environment are not standard and the acquisition and labeling of emotional speech samples requires massive cost, in which the data imbalance of each emotion category appears frequently [18]. In response to such problem, some researchers have processed the data to reduce the degree of imbalance among samples in means of synthesis, screening, and so on [19,20]. The common methods include subsampling and oversampling. The subsampling method is generally applied to the case where the data imbalance is small and the subset of the majority are sufficient, but it causes a certain degree of emotional information loss. Oversampling can reduce the degree of data imbalance at the data level by constructing new samples, but artificially synthesized minority samples may increase the risk of overlearning in minority samples [21]. In addition, some learning models have been improved to reduce the impact of data imbalance on the learning process [22,23,24,25]. Neural network is also a way to deal with data imbalance. R. Alejo et al. improved the BP neural network, combining cost-sensitive learning methods and Gabriel Graph Editing (GGE) to deal with data imbalance [26]. W. Zong et al. proposed a weighted ELM algorithm, which assigns extra weights to each sample to eliminate the impact of imbalance on traditional algorithms [27]. However, due to the long duration of the neural network convergence [26], neural networks have rarely been used to deal with data imbalance in recent years. SMOTE algorithm has been widely used in data imbalance processing, and some improved SMOTE algorithms have been put forward. For example, H. Han et al. proposed the borderline-SMOTE algorithm, which generates synthetic data only for those minority sample data at the decision boundary [28]. H. He et al. proposed the adaptive synthetic sampling approach for imbalanced learning (ADASYN) algorithm, which can adaptively generate different numbers of synthetic data based on data distribution [29]. C. Bellinger et al. proposed a general framework for manifold-based synthetic oversampling that helps users to select a domain-appropriate manifold learning method [30]. However, these algorithms depend too much on the number of nearest neighbors selected, so that some small samples are easily mistaken for noisy data. In SER, unbalanced emotional speech samples often exhibit multi-category, small-scale, and high degree of emotional confusion [31]. At present, only a few data imbalance processing methods have been studied for SER.

One of the major issues of SER is acquiring an optimal emotional feature set from initial signals [3]. Most of the related works have been devoted on the extraction of speech spectral information and prosodic features [32]. In addition, some new feature parameters such as Fourier parameters were used for SER [33]. Most speech emotion features proposed in recent years have been proved to posses emotional representational validity. However, the stability of these features under different speech sample conditions is often not guaranteed [34,35]. Thus, some researchers tend to adopt hybrid emotional feature set containing different categories of features [36]; however, there may exist a great deal of redundant features for different speech samples, which will increase the learning difficulty and computational cost of emotional classifier. Feature selection is indispensible for SER [37,38], in which some linear dimensionality reduction methods have been applied for reducing the dimensionality of speech features [39,40]. In addition, correlation analysis and the wrapped feature selection method that selects the optimal subset in various combinations through different strategies were used in SER [41,42,43].

In summary, there are two main problems with SER in small sample environment. Firstly, data imbalances always exist in the emotional corpora, which impact the learning of different emotional categories in the decision space by the classifiers [44]. Secondly, excessive high-level emotional feature sets have massive redundancy under different sample conditions, which have a great influence on the overall emotional description ability of the feature set.

To solve these problems, a framework of SER in small sample environment is presented to reduce the influence of individual differences and enhance the practicability of SER, in which a selective interpolation synthetic minority over-sampling technique (SISMOTE) is proposed to reduce the imbalance of emotional information. Feature selection based on variance analysis and gradient boosting decision tree (GBDT) is introduced to reduce the feature redundancy. Comparative experiments are performed to verify the effectiveness of the proposed method using CASIA (Chinese emotional corpus) [45], Emo-DB (German emotional corpus) [46], and SAVEE (English emotional corpus) [47]. The unprocessed samples and the samples processed by different methods, i.e., the subsampling algorithm, the random oversampling algorithm, SMOTE algorithm, ADASYN algorithm, borderline-SMOTE algorithm, and SISMOTE algorithm are tested using the same classifier respectively. Different feature selection methods such as Pearson, Random Forest (RF) are compared using standard samples. Furthermore, experiments on speaker-dependent emotion recognition on different databases are performed, which demonstrate better accuracy as compared to state-of-the-art works on the tested databases.

The main contributions of this paper are twofold. Firstly, selective interpolation synthetic minority over-sampling technique (SISMOTE) is proposed to reduce data imbalance, in which the decision space of minority class is expanded as much as possible while reducing the influence of the synthetic samples on the decision space of majority class. Secondly, feature selection based on variance analysis and gradient boosting decision tree (GBDT) is introduced, which could eliminate the redundancy between features and obtain optimal feature set with stronger characterization.

The rest of paper is organized as follows. Feature extraction and data imbalance processing in SER are presented in Section 2. Feature selection of speech features is introduced in Section 3. Experiments on SER and discussion are given in Section 4.

## 2. Data Imbalance Processing in SER

**Figure 1:**
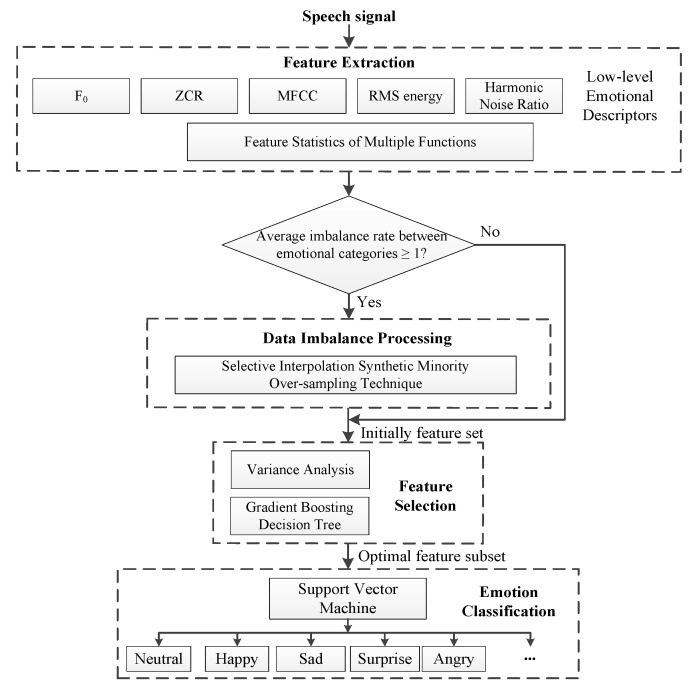
Flowchart of the proposed speech emotion recognition.

Flowchart of the proposed SER model is shown in Figure 1. After obtaining the initially extracted features from preprocessed speech signal samples based on the low-level emotional descriptors (LLEDs) [48,49,50], the remaining procedure mainly consists of three blocks, i.e., data imbalance processing, feature selection, and emotion classification.

As shown in Figure 1, in the actual implementation of the recognition model, it is necessary to calculate the average imbalance ratio of the emotion categories of the initial feature data after feature extraction, thereby determining whether to perform the data imbalance processing on the sample data.

In data imbalance processing, a selective interpolation synthetic minority over-sampling technique (SISMOTE) is proposed for solving the problem of unbalanced data appearing in emotional classification. In feature selection, a new method based on variance analysis and gradient boosting decision tree (GBDT) is introduced to gain the lower redundancy features, in which the variance test as a feature pre-selector can quickly remove redundant features while reducing the calculation of the post-order process and GBDT can obtain the importance rank of emotional features through the fitting of the learner. Support Vector Machine is adopted to classify the emotion categories such as neutral, happy, sad, surprised, and angry.

### 2.1. Deficiency of Traditional SMOTE in Data Processing

The designed samples from standard recorded database are generally used to train models for emotion recognition. However, the observation samples collected in the actual application environment are not ideal and often have sample imbalances which causes recognition problems for machines. For imbalanced data sets, samples of minority class are sparsely distributed in sample space compared with the overwhelming amount of majority class.

**Figure 2:**
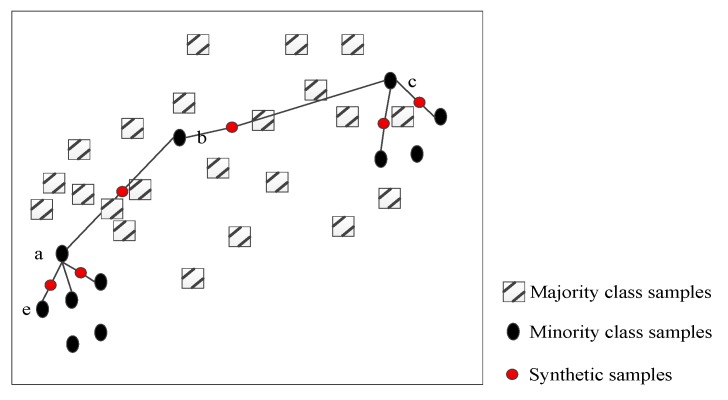
Diagram of the traditional SMOTE algorithm.

Synthetic minority over-sampling technique (SMOTE) is a classical oversampling algorithm that constructs corresponding new samples from minority class information obtained by neighbor relations, which is a scheme based on random oversampling algorithm [51]. The implementation of SMOTE is mainly to find *k* nearest neighbors by Euclidean distance for each sample $x_{i}$ in minority classes, and randomly select a neighbor in the set of neighbors to perform linear interpolation, by which the extension of minority classes is relized. Figure 2 is the schematic diagram of the traditional SMOTE algorithm for two-dimensional feature set. In the example shown in the figure, a few speech sample points from group “a” perform the synthesis of new sample points after linear interpolation of random selections including neighboring points such as “b”, “c”, and “e”. A visual representation of the interpolation process of SMOTE synthesis of minority class in unbalanced emotional speech samples is shown, in which the displayed synthetic sample points are all new samples that may be synthesized.

In spite of synthetic samples being obtained, some shortcomings of the traditional SMOTE may appear. Firstly, the new sample interpolated between sample points “a” and “b” interferes with the decision space of most types of speech samples, and even the new sample may coincide with the majority of samples. The sample point “b” is used as the neighbor of “c”, and the same problem occurs when the sample point “c” is interpolated. Then, the synthesized sample points can only be in the line between them when the sample points “a” and “e” are interpolated.

When SMOTE is applied to emotional speech sample processing, it needs to be improved to overcome these shortcomings. Firstly, it uses all the minority samples in the sampling without considering whether there will be noise data in these samples [28]. Although the sampling space can be expanded to increase the recognition accuracy of the minority class after completing the sampling process, it will affect the decision space and recognition accuracy of majority class. Secondly, it is considered as an interpolation method. If the feature dimension of the sample is two, the new sample $x_{ij}$ synthesized by the algorithm is limited to the line $x_{i}$ is connected to its neighbor point $x_{nn}$. This interpolation method is limited by the way of extension of minority samples.

### 2.2. Imbalance Data Processing Based on SISMOTE

**Figure 3:**
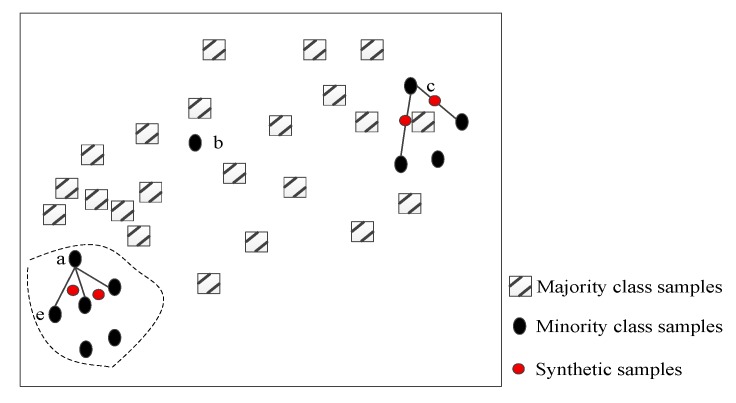
Diagram of the SISMOTE algorithm.

In view of the above analysis, a selective interpolation synthetic minority over-sampling technique (SISMOTE) is proposed to solve the problem of data imbalance in SER, for which not all the minority samples need to be upsampled, but only the corresponding target points are interpolated. Difference from the traditional SMOTE is that the decision space of minority class is expanded as much as possible while reducing the influence of the synthetic samples on the decision space of majority class. Figure 3 is the schematic diagram of the SISMOTE algorithm for two-dimensional feature set.

Combined with the schematic diagram in Figure 3, the SISMOTE can be divided into the following steps.

Step1: For each speech sample in minority emotional classes $x_{i}$, *k* nearest neighbors of that sample are calculated based on Euclidean distance and the set of neighbors is denoted as $S_{i1}$. Besides, *k* nearest neighbors in all speech samples for $x_{i}$ are obtained and its neighbor set is denoted as $S_{i2}$.

Step2: Let $num_{i}=count\left(S_{i1}\cap S_{i2}\right)$. If $num_{i}=0$, $x_{i}$ is marked as a noise point, eliminate it without participating in any subsequent sampling operations. If $0<num_{i}<=k/2+t$, *t* is the regulatory factor, $x_{i}$ is marked as a target point. If $k/2+t<num_{i}<=k$, $x_{i}$ is marked as a non-target point, and mark the $num_{i}$ neighbors of $x_{i}$ as points in the security domain *Q*.

Step3: Count the number of target points *n*, and determine the sampling magnification of the algorithm *N* according to the imbalance ratio of the emotional sample and the number of target points, i.e., N=(M−T)/(T−n), *M* is the number of speech samples of majority emotional class, and *T* is the number of speech samples of minority emotional class.

Step4: Interpolate all target points to construct a new sample. If the target point $x_{o}$ belongs to the security domain, i.e., $x_{o}\in Q$, randomly select two neighbors points ${\tilde{x}}_{1}$ and ${\tilde{x}}_{2}$ from the set of neighbors of $x_{o}$ in minority class samples for one interpolation, i.e., $x_{new}=x_{o}+rand\left(0,1\right)\times \left({\tilde{x}}_{1}-x_{o}\right)+rand\left(0,1\right)\times \left({\tilde{x}}_{2}-x_{o}\right)$, as shown in the dotted line area in Figure 3. On the other hand, randomly select one neighbor point ${\tilde{x}}_{1}$ from the set of neighbors of $x_{o}$ in minority class samples for one interpolation, i.e., $x_{new}=x_{o}+rand\left(0,1\right)\times \left({\tilde{x}}_{1}-x_{o}\right)$.

As shown in Figure 3, a small number of samples is under the same distribution of samples as Figure 2. Sample point “a” chooses the nearest neighbor points to interpolate and construct a few new samples. Sample point “b” is directly judged as noise sample points and no longer participates in any post-order interpolation operations. This helps avoid the influence on decision space of most speech samples. In view of the different neighbor distributions of sample points “a” and “c”, the interpolation space between sample point “a” and nearest neighbor points is no longer available in two-dimensional speech feature space. Limited to the connection line, the region expands to a triangular region, while the sample point “c” still adopts linear interpolation method, i.e., interpolation in the connection area between the sample point “c” and its nearest neighbors due to the small number of neighbors.

At the same time, because SER is usually treated as a multi-classification problem, sampling rate is determined by the most size of emotion categories and the number of other emotion class with the “one-to-many” strategy. For each minority category, other emotion categories are treated as one category, i.e., *l* class data imbalance processing is transformed into l−1 two-class data imbalance processing, so as to achieve multi-class speech data imbalance processing.

## 3. Feature Selection of Speech Features

Importance of each selected feature depends on the correlation between feature and emotional category. The stronger the correlation is, the better the classification ability of representative feature will be. A feature selection method based on variance analysis and gradient boosting decision tree (GBDT) is introduced for SER.

### 3.1. Emotional Feature Pre-Selection Based on Variance Analysis

The idea of variance analysis [52] is to use the divergence indicator to evaluate each feature and select the feature whose score is greater than the threshold. Specifically, the divergence of each feature is calculated, and the feature with the divergence less than the threshold value before the selection is removed. Feature selection is independent of the learner, which is equivalent to filtering the initial features first. In this paper, variance is used as the feature scoring standard. If the difference of the score of a feature is not large, it can be considered that the feature has little contribution to distinguishing the emotional sample. Therefore, the feature with the variance less than the threshold is firstly removed, by which it is possible to achieve rapid feature pre-selection in SER while reducing the feature set dimension and the computational complexity of the subsequent sequence process.

The formula for calculating the variance of each dimension in pre-selection is

(1) $$\delta_{E}^{2}=\frac{\sum_{i=1}^{m}{\sum_{j=1}^{k\times n}\left(x_{ij}-\bar{x_{i}}\right)}^{2}}{m\times k\times n}$$

where *m* is the number of speakers in the emotional speech samples, *k* is the number of emotional categories in the samples, and the *n* is the number of speech samples of each speaker in different emotion categories in the sample set, which is randomly selected.

By setting the threshold of variance, the dimension which has variance less than the threshold is considered to have little contribution in differentiating emotions, therefore such features are removed in the process of constructing the feature set. The pre-selection process of speech emotional features based on variance analysis is independent of the learner, which is equivalent to filtering the initial features and training the learner with the pre-selected feature set.

### 3.2. Importance Evaluation of Emotional Features Based on GBDT

Feature selection based on gradient boosting decision tree (GBDT) is introduced to eliminate the redundancy between features and obtain optimal feature set with stronger characterization. GBDT is integrated learning method based on Boosting strategy [53], in which multiple weak learners with strong dependencies are integrated to obtain the final strong learner through the collection strategy.

The procedure of it can be summarized as the following steps.

Step1: Complete the extraction process of the speech emotional features, setting the maximum number of iterations *T* and the number of leaf nodes *J*.

Step2: Initialize the estimated value of all speech samples for *K* categories, and the following learning and updating process is iteratively performed *T* times.

Step3: Perform a logistic transformation on the function estimates for each speech sample, traversing the probability of each emotional class for all speech samples, and calculating the probability gradient of each speech sample for the *k*th emotional class.

Step4: Obtain the regression tree of *J* leaf nodes through the gradient method, calculating the gain of each leaf node, and updating the estimated values of all speech samples under the *k*th emotional class.

Step5: Keep iterating until the terminating condition is reached, calculating the importance ranking of the emotional feature set through the fitted decision tree group, and obtaining the corresponding feature subsets by setting the importance threshold.

## 4. Experiments

The experiment was designed with the following steps. First of all, openSMILE toolkit and MATLAB R2012b were used to extract speech acoustic features, in which multidimensional features were extracted separately. And the proposed SISMOTE was carried out in data imbalance processing, in which the unbalanced emotional data reaches equilibrium. Then redundancies in emotional data were removed by the model of variance analysis and GBDT. Finally, Support Vector Machine (SVM) [54] was used for speech emotion classification and a Radial Basis Function (RBF) was used as kernel function, in which penalty coefficient *C* and kernel parameter gamma were obtained based on grid search. The experimental process of SER was realized by Python3.5 and MATLAB R2012b program. Experiments were carried out on a 32-bit Windows 7 operating system running on dual-core Intel i5 CPU clocked at 2.4 GHz and using physical memory of 3.16 G.

Using extracted speech features, three sets of experiments were conducted. First, using the same initial feature set, the experiments by different data imbalance processing methods were performed to verify the effectiveness of SISMOTE on Emo-DB and SAVEE. Then, using the same initial emotional feature set and emotional classifier, experiments by different feature selection methods were performed to verify the validity of the proposed feature selection method on CASIA, SAVEE, and Emo-DB databases. Finally, speaker-dependent SER on CASIA, SAVEE and Emo-DB databases was carried out, in which our method was compared with some state-of-the-arts works. To ensure the rigor and fairness of the experiment, the average results are obtained after running experiments ten times.

### 4.1. Speech Database

#### 4.1.1. CASIA Chinese Emotion Corpus

It is recorded by the Institute of Automation, Chinese Academy of Sciences. It has 9600 short mandarin utterances, in which 6 emotional states (i.e., sad, angry, fear, surprise, happy, and neutral) are contained in total, and the emotional samples of this database is recorded by four speakers (i.e., 2 males and 2 females) in a noise-free setting with 16-bit PCM WAV at 16 kHz sampling frequency with about 35 dB [45].

#### 4.1.2. Surrey Audio-Visual Expressed Emotion (SAVEE) Database

It consists of 480 short English utterances recorded by four speakers in seven basic emotions (i.e., angry, fear, disgust, surprise, happy, sad, and neutral), in which the speech samples are picked from the standard TIMIT corpus and each emotion is phonetically-balanced [47].

#### 4.1.3. Berlin Database of Emotional Speech (Emo-DB)

It is a German emotional speech database recorded by the Technical University of Berlin, by the 10 actors (5 males and 5 females) of 10 statements (5 long 5 short) of seven emotions (i.e., happy, angry, anxious, fearful, bored, disgusted, and neutral) simulation, contains a total of 800 sentence corpus, sampling rate of 48 kHz (16 kHz, 16 bit after compression) [46]. The speech recorded in a professional studio, and the actors were required to interpret particular emotion before through the memories of their true experience or experience of mood brewing, to enhance the sense of reality of emotions.

### 4.2. Emotional Data Imbalance Processing

The SISMOTE was tested using standard feature set INTERSPECH 2010 [50] on the Emo-DB and SAVEE database because 2–5 times imbalance in the data of each emotional category exist, and seven sets of experiments were performed using different kinds of data imbalance processing method in total (i.e., None, Subsampling, Random Oversampling, SMOTE, ADASYN, borderline-SMOTE, and SISMOTE). The original sample feature set and the feature set processed by different kinds of data imbalance processing method were used in SD SER experiment respectively. SVM was used for emotion classification.

All samples of each individual were used, in which 70% samples were randomly used for training and the remaining 30% samples were used for testing. The experiment was divided into two groups based on different databases. The first group, i.e., 535 emotional speech samples in Emo-DB were randomly divided into training set and testing set in proportion (7:3), in which the emotional samples in training set were divided into 90 “anger” samples, 58 “boredom” samples, 30 “disgust” samples, 56 “anxiety” samples, 56 “happiness” samples, 36 “sadness” samples, and 36 “neutral” samples. After these unbalanced training samples were processed in different ways and training the SVM classifier, 161 test sets were used to test the classifier. The second group, i.e., 480 emotional speech samples from SAVEE were randomly divided into 336 speech samples in training set and 144 speech samples in testing set, in which the training set consists of 43 “anger” samples, 44 “disgust” samples, 38 “fear” samples, 42 “happiness” samples, 79 “neutral” samples, 48 “sadness” samples, and 42 “surprise” samples. Table 1 shows the comparative results in the initial samples and the samples after using SISMOTE on Emo-DB. Table 2 gives the comparative results in the initial samples and the samples after using SISMOTE on SAVEE.

As shown in Table 1, through the unbalanced data processing, the accuracy and recall rate of the “angry” category are 0.76 and 0.95, respectively. The former represents 76% of all the samples identified by the learner as the category, 24% is actually other categories; the latter shows that 95% of the test samples in this category are correctly classified, and 5% of the samples are misclassified into other emotional categories.

The Emo-DB’s 374-sentence training set samples were processed in five different ways for unbalanced emotional speech. After using up-sampling method, the training set samples included 210 sentences. The training set samples were expanded to 630 sentences after using other over-sampling methods and the speech samples was balanced among emotional classes. In the same way, SAVEE’s 336 speech samples were processed by different methods. The training set samples were reduced to 266 sentences after using up-sampling method and the training set samples were expanded to 553 sentences after using other over-sampling methods.

The classification model was trained using the processed data, and the test was performed using the same testing set. Comparative results using different data imbalance methods on Emo-DB and SAVEE are shown in Table 3.

The average imbalance rate of data between the Emo-DB and SAVEE training episodes of emotional speech sample categories exceeds 100%. Table 1 shows the emotion recognition results when no data imbalance is processed including the accuracy of each emotion category in the two sets of data. Both the precision and the recall are greatly offset, and the corresponding F1 value is lower, e.g., the precision of “happy” category on Emo-DB is 0.87, which indicates that 87% of the results are correctly classified, and the recall rate is only 0.52, which means that the classifier only classifies 52% of the test samples of the category correctly, which results in an overall F1 value being only 0.62. This shows that the data imbalance between the categories of speech data extremely affects the learning of sentiment classifiers. Excessive attention to most types of speech samples leads to higher recall rates and relatively lower accuracy in most emotional categories, such as “angry” and “neutral” in Table 1, while the under-learning of a few categories leads to a lower recall rate and higher accuracy, which affects the overall emotional recognition accuracy.

As shown in Table 1 and Table 2, the precision and the recall rate of each emotional category are lower than that of the data unbalanced processing, and the F1 values of each category are given, from which the data imbalance processing method improves recognition results obviously. The degree of improvement indicates that data for different emotion categories in the training set are balanced and supplemented, and the learner’s degree of over-learning for most types of emotion categories and the degree of under-learning for a few classes are greatly reduced.

At the same time, the recognition accuracy of the learning model by different data imbalance processing methods in Table 3 demonstrates the effectiveness of the SISMOTE algorithm for unbalanced emotional speech data compared to other methods. The emotional data imbalance processing method can extend the decision space of a few sentiment categories to achieve the inter-class balance while reducing the influence of the synthesis of minority speech samples on the decision space of most emotional classes.

### 4.3. Speech Emotional Feature Selection

In this section, the emotional feature selection method based on variance analysis and GBDT was tested using standard feature set INTERSPEECH 2010 on CASIA, Emo-DB, and SAVEE databases, in which the comparison among three feature selection methods (i.e., Pearson correlation analysis, RF, and our method) by seven kinds of classifiers (i.e., Naive Bayes classifier (NB), K-NearestNeighbor (KNN), Logistic Regression (LR), Decision Tree (DT), and SVM) was performed.

The experiments were divided into three groups according to the source of the emotional corpus. The first group has 5400 training samples and 1800 test samples after dividing the data of CASIA. Because the CASIA data are standard and the data imbalance rate among the emotional categories of feature set is lower, the feature selection process was directly performed on the initial feature set without data imbalance processing.

In the initial emotional feature set of CASIA speech sample data (1582 dimensions), the training set was processed by different feature selection methods. The average dimension of the feature set after variance analysis based feature pre-selection is 1253 dimensions, in which the threshold of variance is set to be 0.001. The average dimension of the GBDT importance assessment completion feature selection is 292 dimensions; the average dimension after RF feature selection is 230 dimensions. Different classifiers (i.e., NB, KNN, LR, DT, SVM) were trained using the feature subsets generated by different feature selection methods, and the testing sets were aligned at the same time. Table 4 shows the recognition results using proposed method on CASIA. Table 5 shows the average recognition accuracy of speaker-dependent emotion recognition on CASIA. Table 6 shows the UAR of speaker-dependent emotion recognition on CASIA.

The second group has 401 training samples and 134 testing samples after dividing the data of Emo-DB. The training set samples were expanded to 679 sentences after the data imbalance processing. Same as above, originating from the initial sentiment feature set of Emo-DB speech sample data (1582 dimension), the average number of dimensions for pre-selected feature set selected by variance analysis is 1217, and the average dimension after the GBDT feature selection is 259 dimensions. The average dimension after RF feature selection is 283 dimensions. Table 7 shows the recognition results using proposed method on Emo-DB. Table 8 shows the average accuracy of speaker-dependent speech emotion recognition on Emo-DB. Table 9 shows the UAR of speaker-dependent speech emotion recognition on Emo-DB.

The third group has 360 training samples and 120 test samples after dividing the data of SAVEE. After the data imbalance processing, the training set samples were expanded to 609 sentences. Same as above, originating from the initial sentiment feature set of SAVEE speech sample data (1582 dimension), the average number of dimensions for pre-selected feature set selected by the variance analysis is 1137, and the average dimension after the feature selection is 303 dimensions based on the gradient lifting tree importance evaluation, the average dimension after RF feature selection is 313 dimensions. Table 10 shows the recognition results using proposed method on SAVEE. Table 11 shows the average recognition accuracy of speaker-dependent emotion recognition on SAVEE. Table 12 shows the UAR of speaker-dependent emotion recognition on SAVEE.

For the speech samples under different data distributions, the emotional representation ability of the emotional feature set is improved after the feature selection. As shown in Table 4, “neutral” is identified with the highest accuracy of 96%, and other emotions are classified with accuracies higher than 80% on the CASIA database. Table 7 shows that the precision of “anxiety” and “sadness” reaches to 100% on Emo-DB database. Table 10 shows that “fear” and “neutral” are identified with precision of 88% and 86% respectively, while the precision of the other four emotions does not exceed 80% on SAVEE database. Compared with the experiments using the initial feature set, the proposed feature selection method improves the efficiency while greatly reducing the feature set dimension. The recognition results compared with other feature selection methods demonstrate the effectiveness of the speech sentiment feature selection method based on variance analysis and GBDT.

Table 5, Table 8 and Table 11 show the recognition accuracy using different feature selection method and different classifier on CASIA, Emo-DB and SAVEE database. It shows that our method obtains higher accuracy than other feature selection methods, especially the performance using SVM improves by 5.83% on the SAVEE database.

Unweighted average recall (UAR) is a great evaluation index if various types of emotions have an imbalance distribution [10]. Table 6, Table 9 and Table 12 show the UAR using different feature selection methods (i.e., None, Pearson, RF, and our method) and different classifiers (i.e., NB, KNN, DT, LR, and SVM) on CASIA, Emo-DB and SAVEE database, respectively. It can be seen that our feature extraction method achieves the optimal results using almost all classifiers. In the case of using the SVM classifier, it achieved the optimal UAR on CASIA, Emo-DB, and SAVEE, which are 89.38%, 85.04%, and 74.85%, respectively.

### 4.4. Effect of the Number of Features on SER

In this section, the proposed method was tested using standard feature set INTERSPEECH 2010 on CASIA database, in which the recognition accuracy using different numbers of samples by SVM classifier were given.

**Figure 4:**
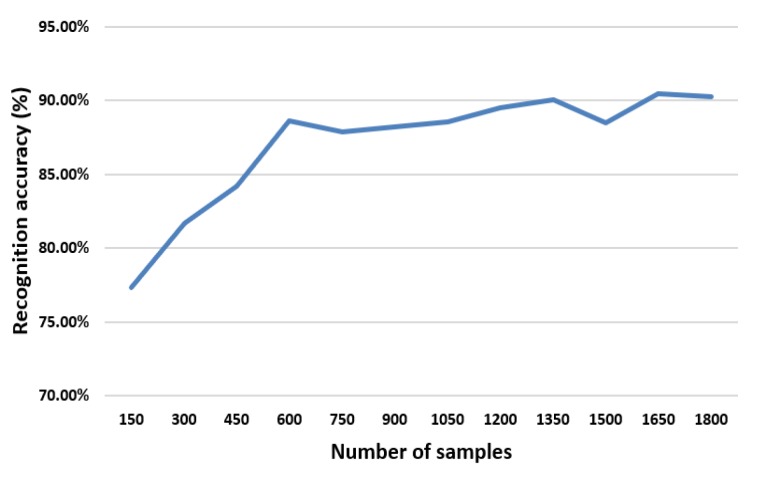
Diagram of recognition accuracy for different sample sizes.

As seen in Figure 4, when the number of samples is less than 600, the recognition accuracy increases along with the increase of sample size. While the sample size exceeds 600, the recognition accuracy is close to 90% and tends to be stable.

### 4.5. Comparison with Some State-of-the-Art Methods

In this experiment, to verify the performance of our method, comparison between our method (SVM is used as the emotional classifier) and some state-of-the-arts methods was carried out. The average recognition accuracy of the proposed speech emotion recognition method on emotional databases is collated, in which our method obtains average recognition accuracy of 90.28% (CASIA), 75.00% (SAVEE), 85.82% (Emo-DB) for speaker-dependent speech emotion recognition. Comparative results of recognition accuracy is shown in Table 13, from which a specific sample environment is modeled in this paper.

As shown in Table 13, our method achieved higher emotion recognition accuracy in SD SER than others. According to the comparison above, our method obtains better results in the case of small sample owing to two main reasons. Firstly, the information of minority emotional class is supplemented while reducing the degree of data imbalance through the SISMOTE. Secondly, feature selection based on variance analysis and GBDT obtains the order of importance of various speech features in emotion recognition through the fitting of the learner, thereby effectively screening the features.

## 5. Conclusions

In this paper, a new framework of SER in small sample environment was put forward, in which the data imbalance processing method based on the selective interpolation synthetic minority over-sampling technique (SISMOTE) in small sample environment was proposed. The effectiveness of the proposal was respectively validated in multiple comparative experiments under different experimental conditions. The SISMOTE was demonstrated to be more suitable for solving data imbalance in speech emotion recognition than the traditional SMOTE. The utility of the feature selection based on variance analysis and GBDT was verified through the experimental comparison.

In future work, the optimization of the model under different conditions will be carried out. For example, the sample size of the database used in this study is not large. If the data size of an unbalanced sample set is large, the distribution of samples in emotional categories may get complicated. The target domain division of a few sentimental classes may be affected, thus affecting the quality of synthesis minority emotional samples. In view of the data imbalance problem in the large sample environment, it is worthwhile to improve the existing algorithms or develop new ones.

In addition, the robustness of the proposed method will be studied. More situations such as noisy environment and cross language environment will be concerned. Furthermore, data preprocessing and feature selection are two indispensable steps for both soft classification and regression. Since SISMOTE belongs to the data preprocessing and another method (i.e., variance analysis and GBDT) belongs to the feature selection, we believe that our method can be applied to both soft classification and regression in the future. To improve the applicability of our method further, we will study on speaker-independent (SI) speech emotion recognition. Since SER is a promising work, the proposal can be applied to many occasions such as advanced driver assistant system (ADAS), remote education, human-robot interaction (HRI). The proposed method in this paper will be applied to the multi-modal emotion recognition system [17] and communication atmosphere modeling in human-robot interaction [58].

## Figures and Tables

**Table 1:** Comparative results in the initial samples and the samples after using SISMOTE on Emo-DB.

Category	None			SISMOTE			Count
	Precision	Recall	F1	Precision	Recall	F1	
Anger	0.76	0.95	0.84	0.76	0.95	0.84	37
Boredom	0.70	1.00	0.82	0.77	1.00	0.87	23
Disgust	0.92	0.75	0.83	1.00	0.75	0.86	16
Anxiety	0.64	0.69	0.67	0.75	0.69	0.72	13
Happiness	0.87	0.52	0.65	0.94	0.64	0.76	25
Sadness	0.86	0.83	0.84	0.84	0.91	0.87	23
Neutral	0.83	0.62	0.71	0.89	0.71	0.79	24
Avg/Total	0.80	0.78	0.78	0.84	0.83	0.82	161

**Table 2:** Comparative results in the initial samples and the samples after using SISMOTE on SAVEE.

Category	None			SISMOTE			Count
	Precision	Recall	F1	Precision	Recall	F1	
Anger	0.50	0.65	0.56	0.57	0.71	0.63	17
Disgust	1.00	0.31	0.48	1.00	0.38	0.55	16
Fear	0.82	0.41	0.55	0.81	0.59	0.68	22
Happiness	0.50	0.50	0.50	0.71	0.56	0.63	18
Neutral	0.74	0.98	0.84	0.82	0.98	0.89	41
Sadness	0.69	0.75	0.72	0.60	0.75	0.67	12
Surprise	0.62	0.72	0.67	0.65	0.83	0.73	16
Avg/Total	0.70	0.67	0.65	0.76	0.73	0.72	144

**Table 3:** Comparative results using different imbalance processing methods on Emo-DB and SAVEE.

Database	Recognition Accuracy (%)						
	None	Subsampling	Random Oversampling	SMOTE	ADASYN	Borderline-SMOTE	SISMOTE
Emo-DB	78.26%	74.53%	81.75%	81.99%	82.09%	82.15%	82.61%
SAVEE	66.67%	57.64%	70.14%	72.22%	72.50%	72.53%	72.92%

**Table 4:** Recognition results using proposed method on CASIA.

Category	Precision	Recall	F1	Count
Anger	0.94	0.92	0.93	296
Fear	0.90	0.84	0.87	300
Happy	0.86	0.90	0.88	304
Neutral	0.96	0.95	0.96	288
Sadness	0.84	0.91	0.87	288
Surprise	0.93	0.91	0.92	324
Avg/Total	0.90	0.90	0.90	1800

**Table 5:** Average recognition accuracy of speaker-dependent emotion recognition on CASIA.

Classifier	Recognition Accuracy (%)			
	None	Pearson	RF	Our Method
NB	44.22	44.39	50.89	50.39
KNN	62.00	59.83	76.83	74.72
DT	59.83	55.00	62.83	62.11
LR	81.17	78.00	82.44	84.39
SVM	88.39	84.78	88.61	90.28

**Table 6:** Unweighted average recall of speaker-dependent emotion recognition on CASIA.

Classifier	Unweighted Average Recall (%)			
	None	Pearson	RF	Our Method
NB	44.39	44.76	51.23	52.26
KNN	62.08	59.98	78.11	78.90
DT	59.13	53.93	61.15	63.27
LR	81.16	78.04	82.29	82.78
SVM	88.42	84.81	88.69	89.38

**Table 7:** Recognition results using proposed method on Emo-DB.

Category	Precision	Recall	F1	Count
Anger	0.87	0.87	0.87	30
Boredom	0.83	0.83	0.83	18
Disgust	0.78	0.90	0.84	20
Anxiety	1.00	0.92	0.96	12
Happiness	0.78	0.78	0.78	22
Sadness	1.00	0.81	0.90	16
Neutral	0.86	0.90	0.88	20
Avg/Total	0.80	0.86	0.86	134

**Table 8:** Average recognition accuracy of speaker-dependent speech emotion recognition on Emo-DB.

Classifier	Recognition Accuracy (%)			
	None	Pearson	RF	Our Method
NB	74.63	74.86	73.13	79.34
KNN	64.18	67.16	69.40	72.39
DT	56.72	50.00	52.99	59.70
LR	77.61	80.06	81.34	76.12
SVM	82.09	81.34	83.58	85.82

**Table 9:** Unweighted average recall of speaker-dependent speech emotion recognition on Emo-DB.

Classifier	Unweighted Average Recall (%)			
	None	Pearson	RF	Our Method
NB	73.12	73.93	74.46	80.06
KNN	67.08	69.88	69.19	76.07
DT	57.90	43.77	56.27	57.76
LR	77.46	79.78	81.33	80.52
SVM	81.53	81.53	83.27	85.04

**Table 10:** Recognition results using proposed method on SAVEE.

Category	Precision	Recall	F1	Count
Anger	0.56	0.71	0.63	14
Disgust	0.73	0.57	0.64	14
Fear	0.88	0.74	0.80	19
Happiness	0.69	0.56	0.62	16
Neutral	0.86	0.94	0.90	33
Sadness	0.67	0.75	0.71	8
Surprise	0.71	0.75	0.73	16
Avg/Total	0.76	0.75	0.75	120

**Table 11:** Average recognition accuracy of speaker-dependent speech emotion recognition on SAVEE.

Classifier	Recognition Accuracy (%)			
	None	Pearson	RF	Our Method
NB	55.83	52.50	60.00	60.83
KNN	50.00	50.00	54.17	59.17
DT	43.33	49.17	45.83	46.67
LR	64.17	63.33	65.00	66.67
SVM	69.17	67.5	74.17	75.00

**Table 12:** Unweighted average recall of speaker-dependent speech emotion recognition on SAVEE.

Classifier	Unweighted Average Recall (%)			
	None	Pearson	RF	Our Method
NB	48.78	48.78	57.39	61.11
KNN	54.64	54.64	57.29	58.32
DT	42.47	45.21	49.40	40.07
LR	60.10	60.10	62.31	65.14
SVM	63.87	63.87	67.43	74.85

**Table 13:** Comparison between our method and some related works.

Database	Reference	Average Recognition Accuracy (%)
CASIA	[55]	85.08
	[42]	89.6
	Our method	90.28
SAVEE	[56]	61.25
	Our method	75.00
Emo-DB	[57]	80.5
	Our method	85.82
